# Bafetinib Suppresses the Transcription of PD-L1 Through c-Myc in Lung *Cancer*

**DOI:** 10.3389/fphar.2022.897747

**Published:** 2022-06-02

**Authors:** Xi Chen, Qianqian Du, Hongjie Guo, Qiaojun He, Bo Yang, Ling Ding

**Affiliations:** ^1^ Zhejiang Province Key Laboratory of Anti-Cancer Drug Research, Institute of Pharmacology and Toxicology, College of Pharmaceutical Sciences, Zhejiang University, Hangzhou, China; ^2^ The Innovation Institute for Artificial Intelligence in Medicine, Zhejiang University, Hangzhou, China

**Keywords:** bafetinib, c-Myc, PD-L1, transcription, lung cancer

## Abstract

Given the limitations of the existing antibody-based therapies, including immune-related adverse events, poor response rates, and intravenous route of dosing, small molecules inhibitors targeting PD-L1 are highly desirable. By cell-based screening, we found that tyrosine kinase inhibitor Bafetinib dramatically suppresses PD-L1 protein expression in a dose-dependent manner. In parallel, cell membrane PD-L1 is also reduced by Bafetinib. We confirm that Bafetinib doesn’t affect the protein half-life of PD-L1 but significantly inhibits the transcription of PD-L1. Among the transcription factors that regulate PD-L1 expression, c-Myc is downregulated by Bafetinib. Bafetinib caused PD-L1 inhibition is abolished when c-Myc is knocked-down. Further, we identified that Bafetinib reduced c-Myc expression because of transcription inhibition. By using the CT26 tumor model, we further confirm that Bafetinib suppressed PD-L1 expression *in vivo*. In conclusion, our study shows that Bafetinib inhibits the transcription of PD-L1 through transcription factor c-Myc, suggesting that Bafetinib might be a small molecule drug targeting PD-L1.

## 1 Introduction

The combination of Programmed death-1 (PD-1) and its ligand programmed death ligand-1 (PD-L1) forms an immunosuppressive microenvironment and plays a key role in tumor immune escape (Jiang et al., 2019). At present, many monoclonal antibody drugs based on blocking PD-1/PD-L1 binding have achieved significant therapeutic effects, especially in melanoma and non-small cell lung cancer. Some patients can even reach the state of “clinical cure”, which is a breakthrough in the field of tumor immunotherapy (Pardoll, 2012). However, PD-1/PD-L1 monoclonal antibodies face great challenges. Firstly, the response rate of PD-1/PD-L1 monoclonal antibodies needs to be further improved. In the treatment of most solid tumors, the response rate does not exceed 20% (Brahmer et al., 2012). Some studies believe that one of the reasons is that macromolecular antibody drugs are difficult to penetrate the tumor tissue effectively and can’t reach all regions of the tumor in a sufficient amount. Then, adverse reactions need to be further reduced. Immune-related adverse events caused by antibody therapy are discrete toxicity caused by nonspecific activation of the immune system, which can affect almost any organ system. Compared with macromolecular antibody drugs, small molecule drugs usually have the advantages of good permeability to organs or tumors, less stimulation to the immune system, and oral administration (Adams et al., 2015). Currently, some studies have shown that small molecule inhibitors can inhibit the expression of PD-L1 and play an anti-tumor role. Sp600125 inhibits histone H3 acetylation of PD-L1 promoter through COP1/c-Jun/HDAC3 axis, thereby reducing the transcriptional level of PD-L1 (Wang et al., 2020). Ms-444 down-regulates the expression of PD-L1 by inhibiting HuR, which binds to and stabilizes CMTM6 mRNA and reduces the CMTM6-mediated stabilization of cell-surface PD-L1 (Liu et al., 2021). JQ1 inhibits the transcription of PD-L1 (Zhu et al., 2016). eIF4E inhibits protein translation of PD-L1 (Xu et al., 2019). Curcumin promotes PD-L1 ubiquitination degradation (Lim et al., 2016). However, most of these compounds are experimental tool drugs, which can’t realize the transformation of clinical application. Therefore, looking for small molecule drugs targeting PD-1/PD-L1 for clinical use is considered to be an important way to overcome the defects of antibody drugs, which has become a research hotspot in this field.

Based on the above reasons, we found that Bafetinib (INNO-406, NS-187) can significantly inhibit the expression of PD-L1 by cell-based screening. Bafetinib is a dual Bcr-Abl/Lyn tyrosine kinase inhibitor. Although Bafetinib was originally designed for the treatment of Bcr-Abl^+^ leukemia, including chronic myelogenous leukemia (CML) and Philadelphia^+^ acute lymphoblastic leukemia (ALL) (Kimura et al., 2005), it has been found in subsequent studies that Bafetinib has therapeutic potential in solid tumors such as glioblastoma multiforme (Prakash et al., 2012), malignant melanoma (Zhang et al., 2019), and breast cancer (Tabaries et al., 2015). Up to now, the effect of Bafetinib on the immune microenvironment is not elucidated. Therefore, we explore the mechanism of Bafetinib regulating PD-L1, which may become a potential and effective small molecule inhibitor of anti-PD-L1 immunotherapy in NSCLC. In conclusion, our study showed that Bafetinib reduces the transcription of PD-L1 by inhibiting the mRNA level of its transcription factor c-Myc.

## 2 Materials and Methods

### 2.1 Antibodies and Reagents

The antibodies against PD-L1 (#13684S), CD276 (#14058S), CD47 (#63000S), p-STAT3 (Y705) (#9145S), p-STAT1 (Y701) (#9167S), p-STAT1 (S727) (#8826S) and Galectin-9 (#54330S) were purchased from Cell Signaling Technology (Beverly, ME, United States). Antibodies against STAT1 (ET1606-39), p-NF-κB p65 (S529) (ET1604-27), p-c-Myc (S62) (ET1609-64), p-c-Myc (T58) (ET1611-24), c-Myc (RT1149) were purchased from HuaBio (Hangzhou, China). Anti-STAT3 (10253-2-AP) was purchased from Proteintech (Proteintech, Chicago, IL, United States). Anti-NF-κB p65 (sc-8008) was from Santa Cruz Biotechnology (Santa Cruz, CA). Anti-GAPDH (db106) was purchased from diagbio (Hangzhou, China). Bafetinib (T6311), MG132 (T2154), Chloroquine (T8689), and CMC-Na (T19232) were purchased from TargetMol (Boston, MA, United States). FITC-conjugated CD274 mouse monoclonal antibody (#558065) and FITC mouse IgG were purchased from BD Biosciences (San Jose, California, United States).

### 2.2 Cell Culture

All the cell lines were purchased from the Cell Bank of Shanghai Institutes for Biological Sciences, Chinese Academy of Sciences (Shanghai, China). NSCLC cell lines H1299, H292, H460, H358, PC9, and colon adenocarcinoma cell CT26 were cultured in RPMI-1640 (Gibco, #31800, Gaithersburg, MD, United States) supplemented with 10% fetal bovine serum (FBS; Hyclone, SV30160.03, GE Healthcare, Logan, UT, United States). We maintained all cells in the medium containing 1% penicillin and 1% streptomycin (Gibco, #15140122) and in a 5% CO_2_ incubator at 37°C. All the cell lines were authenticated by short tandem repeat (STR) DNA fingerprinting, most recent authentication on 15 September 2020. All cell lines were tested and were free of *mycoplasma* contamination.

### 2.3 Western Blot

The protein samples of different molecular weights were separated by 10% SDS-PAGE gel electrophoresis, and then they were transferred to polyvinylidene difluoride (PVDF) membranes (Millipore, Bedford, MA, United States). The membranes were blocked with 5% skim milk and incubated with the corresponding primary antibodies at 4°C overnight. Then the membranes were incubated with secondary antibody (1:5,000) at room temperature for 1 h, and the protein bands were analyzed by chemiluminescence with an ECL detection reagent.

### 2.4 Flow Cytometry

H292 cells were collected and washed with PBS. Then the cells were stained with FITC-conjugated CD274 and incubated at 4°C for 2 h. After washing with PBS, the cells were resuspended in 500 μL PBS. The expression of PD-L1 on the surface of the cell membrane was measured by Beckman Coulter flow cytometry (Beckman, DxFLEX, Krefeld, Germany).

### 2.5 siRNA-Mediated Silencing

H292 cells (1.5 × 10^5^) were seeded in 6-well plates for 24 h. Then, cells were transfected with transfection reagent JetPRIME (Polyplus-transfection, #114–15, Illkirch, France), Jet PRIME Buffer (Polyplus-transfection, #712–60), and c-Myc siRNA or siRNA-negative control (Jet PRIME Buffer (Polyplus-transfection, #712–60) 200 μL, JetPRIME 2 μL, 20 μM siRNA 2.5 μL for per well) for 24 h (Ding et al., 2019). The siRNA sequences used in the study are provided in Supplementary Table S1.

### 2.6 Ribonucleic Acid Isolation and Quantitative Real-Time Polymerase Chain Reaction

Total RNA was extracted with Trizol reagent (Invitrogen, #15596026, Waltham, MA, United States) and further purified according to the standard protocol. Single-stranded cDNA was synthesized using TransScript One-Step gDNA Removal and cDNA Synthesis SuperMix (TransGen Biotech, #AT311-03, Beijing, China). Quantitative RT-PCR is completed by SYBR-Green Kit (Bio-Rad, #172–5,124, Richmond, California, United States), and its accuracy can be judged by melting curves and Repeated samples. β-Actin is used as a normalized gene, and the calculation of data needs to be normalized to its mRNA level (Pan et al., 2020). The primers used are provided in Supplementary Table S2.

### 2.7 *In Vivo* Study

5 × 10^5^ murine colorectal adenocarcinoma CT26 cells were subcutaneously inoculated into Male Balb/c mice (6 weeks) or Male immunodeficient nude mice. The mice were randomly divided into two groups, whereby they were treated with 5% CMC-Na or Bafetinib (30 mg/kg daily) dispersed in 5% CMC-Na intragastrically. Tumor volume (TV) and mice body weight were measured every other day (formulate TV = length × wide × width). All animal experiments were approved by the Center for Drug Safety Evaluation and Research of Zhejiang University (IACUC-s22-004).

### 2.8 T Cell-Mediated *Cancer* Cells Killing Assay

Fresh lung cancer tissues and Blood samples for peripheral blood mononuclear cells (PBMC) extraction of patients were obtained with the donor’s written consent and approved by Zhejiang *Cancer* Hospital Committee (IRB-2020–324). PBMC were obtained by density gradient centrifugation using Ficoll-Paque solution (GE Healthcare, 17–1,440–02, Uppsala, Sweden). H292 cells were inoculated into 96 well plates (3,000–5,000 cells/well) and then treated with Bafetinib for 24 h. PBMC cells were activated with anti-CD3/CD28/CD2 (50 µL antibody was added to every 2 ml dish) for 3–5 days in advance, and then co-incubated according to the ratio of PBMC: tumor cells 5:1. After 8–12 h, drain the culture solution with a pipette, dye it with crystal violet at room temperature for 30 min, wash it with PBS until there is no floating color, then add 100 µL PBS and take photos with a microscope.

### 2.9 Statistical Analysis

All statistical data were presented as the mean ± Standard Error of Mean (SEM). Students’ t-test and one-way ANOVA were used to determine statistical differences between groups. *p* < 0.05 was considered significant. ***, *p* < 0.001; **, *p* < 0.01; *, *p* < 0.05; n. s: not significant.

## 3 Results

### 3.1 Bafetinib Inhibits the Expression of Immune Checkpoint PD-L1 in Lung Cancer Cells

**FIGURE 1:**
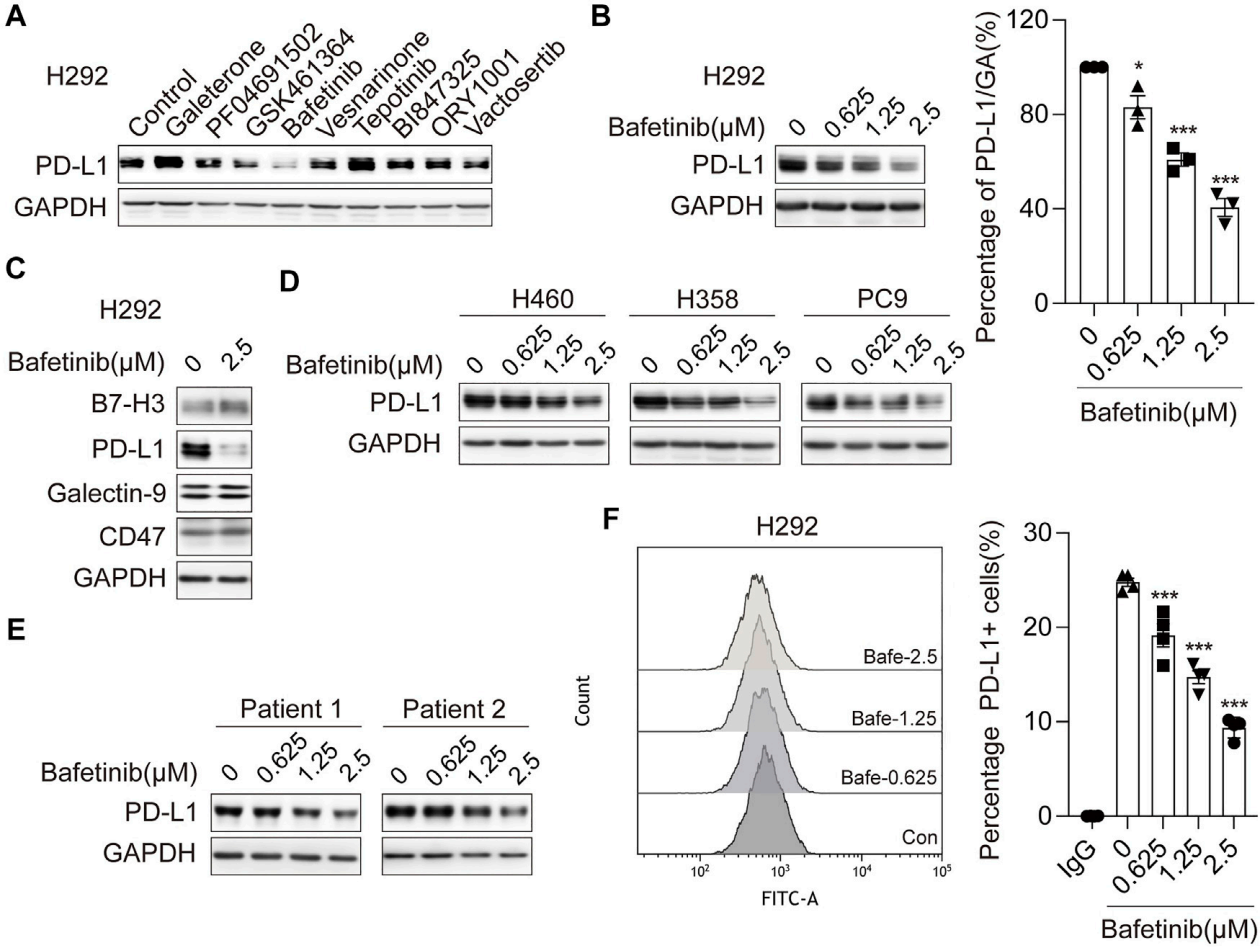
Bafetinib inhibits the expression of PD-L1 in lung cancer. **(A)** Screening on H292 cells treated with different small molecule drugs (10 μM) for 24 h. **(B)** The expression of PD-L1 protein was measured by Western blot in H292 cells, which were treated with Bafetinib (0.625, 1.25, and 2.5 μM) for 24 h. The relative protein level of PD-L1 in H292 was quantitatively analyzed on the right. **(C)** Expression of B7-H3, Galectin-9, PD-L1, and CD47 was measured by Western blot in H292 cells when treated with Bafetinib (2.5 μM) for 24 h **(D, E)** The expression of PD-L1 protein was measured by Western blot in H460, H358, PC9 cells, and primary lung cancer when treated with Bafetinib (0.625, 1.25, and 2.5 μM) for 24 h. **(F)** Surface PD-L1 expression on H292 treated with Bafetinib (0.625, 1.25, and 2.5 μM) was determined by flow cytometry. Cells were estimated for PD-L1 or mouse IgG control antibodies. Data were the mean ± SEM of quadruplicate experiments. The data were analyzed by one-way ANOVA with Dunnett’s post hoc test. ***, *p* < 0.001; *, *p* < 0.05.

To identify small-molecule drugs that suppress PD-L1 expression, we performed a cell-based screening from anti-tumor drugs in NSCLC cell line H292, which displays a relatively high level of endogenous PD-L1. We observed that Bafetinib could significantly inhibit the expression of PD-L1 (Figure 1A and Supplementary Figure S1A). Intriguingly, we found that Bafetinib in the concentration of 1.25 μM (clinical relevant (Shah et al., 2004)) exhibited dramatic inhibition of PD-L1 expression (Figure 1B). To confirm whether Bafetinib selectively affects PD-L1 expression, we analyzed the expression of other immune checkpoints (B7-H3, Galectin-9, and CD47). The data showed that Bafetinib significantly and specifically down-regulated PD-L1 protein in H292 without affecting other immune checkpoints (Figure 1C). At the same time, similar downregulation of PD-L1 was observed on H460, H358, and PC9 (Figure 1D), which was further verified in two cases of primary lung cancer cells (Figure 1E). Considering that cell membrane PD-L1 directly causes the immune evasion of cancer cells, we evaluated cell membrane PD-L1 level after Bafetinib administration. By flow cytometry, we found that Bafetinib reduced the expression level of PD-L1 on the surface of the cell membrane (Figure 1F). Taken together, these data suggested that Bafetinib is a potent small molecule that suppresses PD-L1 expression.

### 3.2 Bafetinib Inhibits the Transcription of PD-L1

**FIGURE 2:**
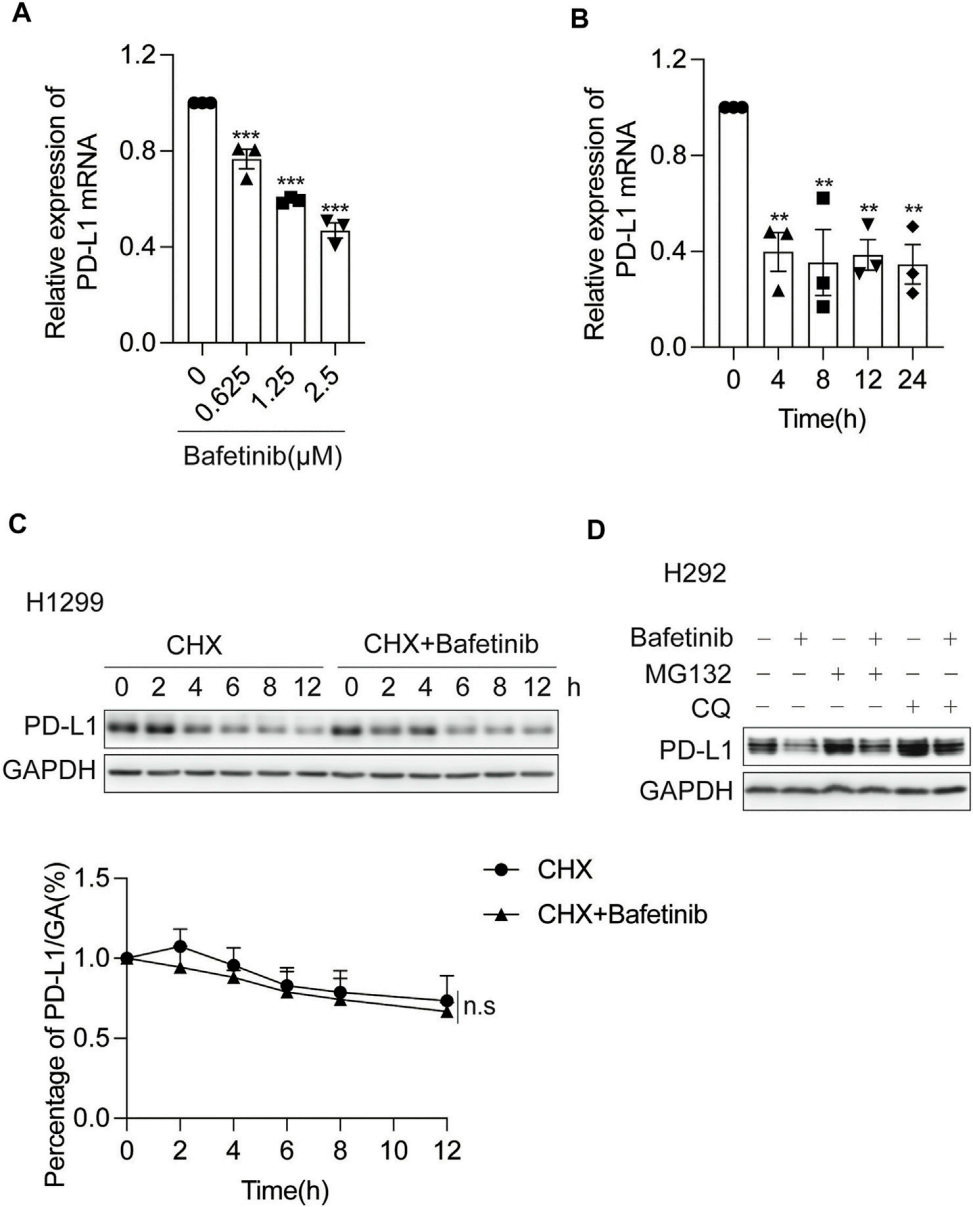
Bafetinib inhibits the transcription of PD-L1. **(A)** H292 cells were treated with Bafetinib (0.625, 1.25, and 2.5 μM) for 24 h, then the relative expression of PD-L1 mRNA was quantified by qRT-PCR *n* = 3. **(B)** H292 cells were treated with Bafetinib (2.5 μM) for 0, 4, 8, 12, and 24 h, then the relative expression of PD-L1 mRNA was quantified by qRT-PCR *n* = 3. **(C)** Western blot of PD-L1 protein in H1299 cells with or without Bafetinib in the presence of CHX at 10 μg/ml for 0, 2, 4, 6, 8, and 12 h. The relative protein level of PD-L1 was analyzed quantitatively below *n* = 3. **(D)** Western blot of PD-L1 protein in H292 cells with or without the treatment of Bafetinib (2.5 μM) meanwhile the cells were treated with MG132 (10 μM) or CQ (20 μg/ml) for 10 h. The data were analyzed by one-way ANOVA with Dunnett’s post hoc test or the Students’ t-test. ***, *p* < 0.001; **, *p* < 0.01; n. s: not significant.

To understand the mechanism of Bafetinib-induced PD-L1 suppression, we measured the mRNA level and protein half-life of PD-L1 in response to Bafetinib treatment. We analyzed the changes in PD-L1 mRNA levels after Bafetinib treatment for 24 h and found that Bafetinib reduced PD-L1 mRNA levels in a dose-dependent manner (Figure 2A). At the same time, our data showed that PD-L1 transcription was significantly reduced in 4 h (Figure 2B). Then, we examined the effect of Bafetinib on the protein half-life of PD-L1 by adding Cycloheximide (CHX), which was used to inhibit protein synthesis (Buchanan et al., 2016) and is widely used to determine the half-life of proteins. As a result, the half-life of PD-L1 is not significantly affected by Bafetinib (Figure 2C). For further confirmation, we treated with cellular ubiquitin-proteasome pathway inhibitor MG132 and lysosomal pathway inhibitor chloroquine (CQ), which are the two main ways of PD-L1 degradation *in vivo* (Dikic, 2017). We found that neither MG132 nor CQ could reverse the down-regulation of PD-L1 by Bafetinib (Figure 2D). These data suggest that Bafetinib significantly inhibits PD-L1 mRNA expression, but did not affect the protein stability of PD-L1.

### 3.3 Bafetinib Inhibits the Expression of PD-L1 by Inhibiting the Transcription of c-Myc

**FIGURE 3:**
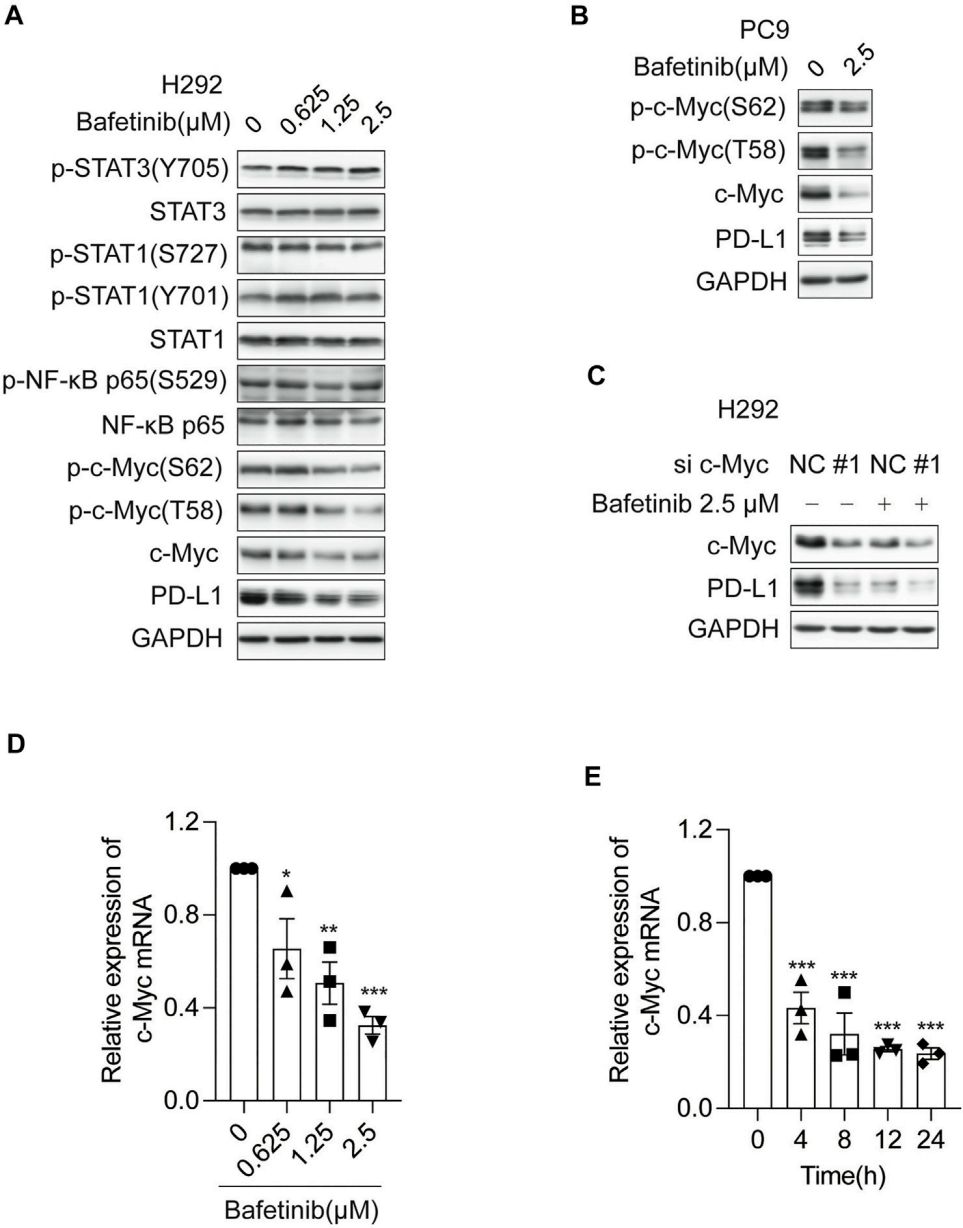
Bafetinib inhibits the expression of PD-L1 by inhibiting the transcription of c-Myc. **(A)** The expression of p-STAT3 (Y705), STAT3, p-STAT1 (S727), p-STAT1 (Y701), STAT1, p-NF-κB p65 (S529), NF-κB p65, p-c-Myc (S62), p-c-Myc (T58), c-Myc and PD-L1 was measured by Western blot in H292 cells after 24 h of Bafitinib (0.625, 1.25 and 2.5 μM) treatment. **(B)** Western blot of p-c-Myc (S62), p-c-Myc (T58), c-Myc, and PD-L1 protein in PC9 cells with the treatment of Bafetinib (2.5 μM). **(C)** The expression of PD-L1 was measured in H292 cells transfected with siRNA-c-Myc (or siRNA-NC) treated with or without Bafetinib (2.5 μM). **(D)** H292 cells were treated with Bafetinib (0.625, 1.25, and 2.5 μM) for 24 h, then the relative expression of c-Myc mRNA was quantified by qRT-PCR *n* = 3. **(E)** H292 cells were treated with Bafetinib (2.5 μM) for 0, 4, 8, 12, and 24 h, then the relative expression of c-Myc mRNA was quantified by qRT-PCR *n* = 3. The data were analyzed by one-way ANOVA with Dunnett’s post hoc test. ***, *p* < 0.001; **, *p* < 0.01; *, *p* < 0.05.

By far, it has been reported that transcription factors including STAT1 (Garcia-Diaz et al., 2017), STAT3 (Song et al., 2018), NF-κB (Bouillez et al., 2017), and c-Myc (Casey et al., 2016) can regulate the transcription of PD-L1 (Yi et al., 2021). To explore which transcription factor was involved in the transcriptional repression of PD-L1 by Bafetinib, we analyzed the changes of each transcription factor and its phosphorylation form after 24 h of Bafitinib treatment on H292 cells. We found that only c-Myc and its phosphorylation form were significantly inhibited (Figure 3A), suggesting that Bafetinib may reduce the expression of PD-L1 by inhibiting the c-Myc pathway. Consistently, a similar down-regulation of c-Myc and its phosphorylation form was observed on PC9 (Figure 3B). In addition, we knocked down c-Myc by siRNA and found that Bafetinib caused PD-L1 reduction was attenuated in the absence of c-Myc (Figure 3C). Besides, we also investigated the regulation of Bafetinib on the transcription level of c-Myc and found that Bafetinib could inhibit the mRNA of c-Myc in a dose-dependent manner (Figure 3D). And c-Myc mRNA reduction was very significant after 4 h of Bafetinib, which overlapped with the suppression of PD-L1 mRNA (Figure 3E). The above data suggest that Bafetinib reduces the expression of PD-L1 by inhibiting the transcription of c-Myc.

### 3.4 Bafetinib Inhibits the Expression of PD-L1 *In Vivo*

**FIGURE 4:**
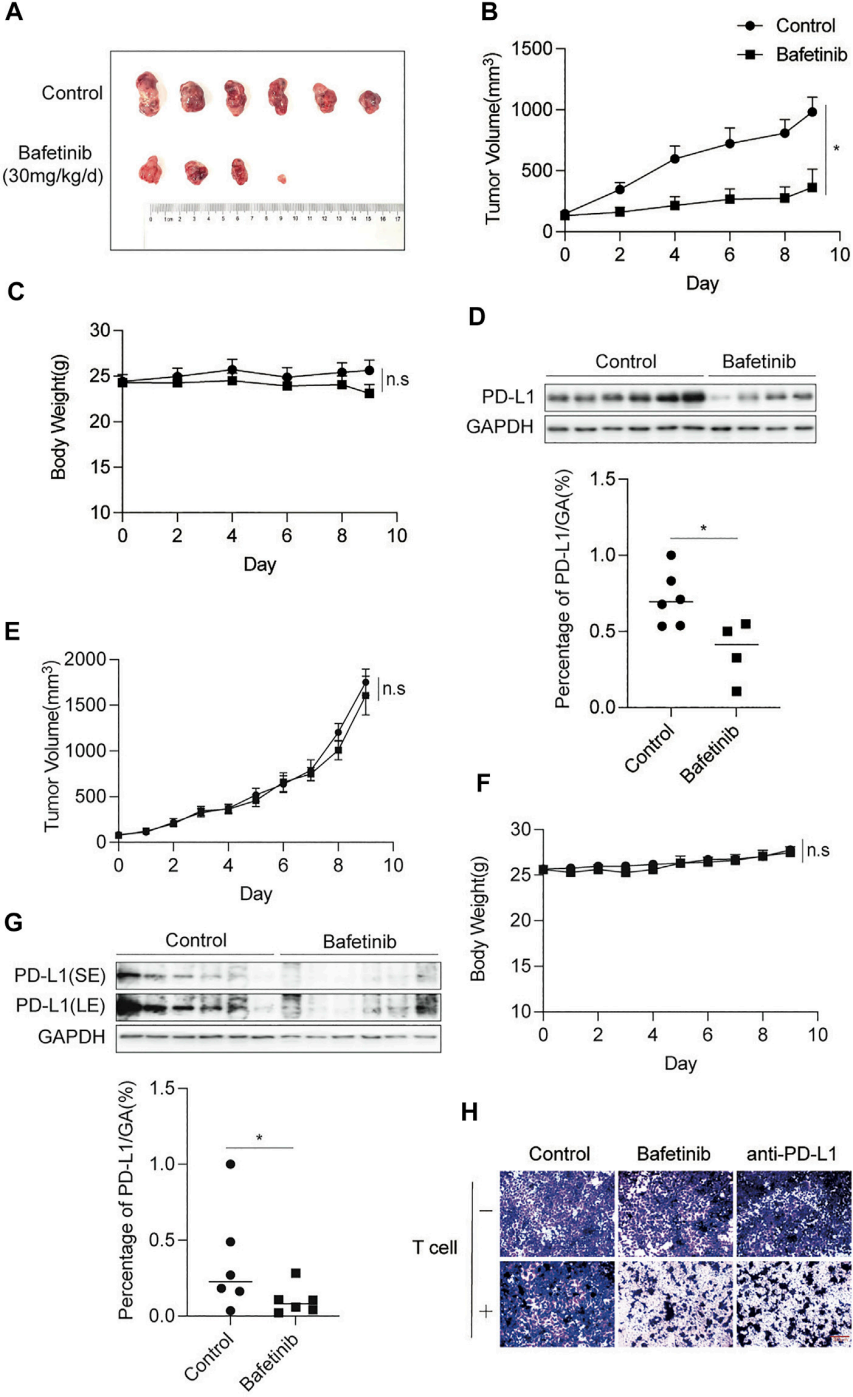
Bafetinib inhibits the expression of PD-L1 *in vivo*. **(A)** Tumor image of Balb/c mice treated with or without Bafetinib (30 mg/kg daily). **(B)** Tumor volume of Balb/c mice treated with or without Bafetinib (30 mg/kg daily). **(C)** The body weights of Balb/c mice were measured every other day. **(D)** Expression of PD-L1 in tumors of Balb/c mice. The relative protein level of PD-L1 in CT26 was quantitatively analyzed below. **(E)** Tumor volume of immunodeficient nude mice treated with or without Bafetinib (30 mg/kg daily). **(F)** The body weights of immunodeficient nude mice. **(G)** Expression of PD-L1 in tumors of immunodeficient nude mice. The relative protein level of PD-L1 in CT26 was quantitatively analyzed below. Bars, mean ± SEM (*n* = 6). *, *p* < 0.05. n. s: not significant. **(H)** H292 cells were treated with control Bafetinib (2.5 μM) or anti-PD-L1. T cells were isolated from peripheral blood and stimulated via anti-CD3/CD28/CD2. Co-incubation was carried out with these treated H292 cells for 8–12 h (T cells: Tumor cells = 5:1). After incubation, surviving cells were then fixed and stained with crystal violet. Sacle bar: 300 μm.

To evaluate the anti-tumor effect of Bafetinib *in vivo*, CT26 cells were inoculated into Balb/c mice, which were given Bafetinib orally at a dose of 30 mg/kg/d or 0.5% sodium carboxymethyl cellulose (CMC-Na) of the same volume for 10 days (Day 0 is the first day of our treatment). C_max_ for Bafetinib was estimated at 661 ng/ml (1.15 μM) when mice were treated once a day orally with 30 mg/kg of Bafetinib (clinically relevant) (Brahmer et al., 2012). Bafetinib treatment significantly inhibited tumor growth compared with the control group (Figures 4A,B). And there was no significant change in the bodyweight of Balb/c mice during Bafetinib treatment (Figure 4C). In addition, compared with the control group, we found that Bafetinib significantly inhibited the expression of PD-L1 in mouse tumors (Figure 4D). The above findings show that Bafetinib can reduce the expression of PD-L1 *in vivo*, to inhibit the occurrence and development of tumors. In addition, we treated immunodeficient nude mouse models bearing CT26 tumors with the same dose of Bafetinib (30 mg/kg/d) for the same time (10 days). Interestingly, Bafetinib didn’t inhibit tumor growth (Figures 4E,F), although we found lower PD-L1 expression in nude mice treated with Bafetinib compared with the control group (Figure 4G). This shows that Bafetinib treatment of tumors depends on the complete immune system. Moreover, we used Bafetinib-treated H292 cells for the T cell killing assay, and we set anti-PD-L1 (50 μg/ml) as a positive control. by using the co-culture system, we further found that Bafetinib enhanced the killing effect of T cells on tumor cells, which suggests that Bafetinib-triggered PD-L1 suppression is sufficient to achieve anti-tumor immunity (Figure 4H).

## 4 Discussion

Because macromolecular antibody drugs have the disadvantages of low response rate and immune-related adverse reactions, looking for small molecule drugs against PD-1/PD-L1 is considered to be an important way to overcome the defects of antibody drugs. Through cell-based screening, we found that Bafetinib, a small molecule tyrosine kinase inhibitor, can significantly reduce the expression of PD-L1 protein in a dose-dependent manner. Similarly, PD-L1 on the cell surface was also reduced by Bafetinib. We demonstrated that Bafetinib inhibited the expression of PD-L1, which didn’t affect the protein stability of PD-L1 but significantly inhibited the transcription of PD-L1. Subsequently, we found that Bafetinib can inhibit the transcription of PD-L1 by inhibiting the transcription of c-Myc. By using the CT26 tumor model, we further confirmed that Bafetinib inhibited the expression of PD-L1 *in vivo*.

The regulation of PD-L1 in the tumor microenvironment is affected by many factors, including genomic alterations, epigenetic modification, transcriptional regulation, post-transcriptional modification, and post-translational modification (Atsaves et al., 2017; Zerdes et al., 2018; Zhu et al., 2018; Yi et al., 2021). Therefore, small-molecule inhibitors can be designed from the aspects of PD-L1 gene transcription, protein translation, and protein post-translational modification to inhibit the expression of PD-L1. It is proved that the small molecule inhibitors of these regulatory proteins can effectively reduce the level of PD-L1 protein and play an anti-tumor role. JQ1 inhibits Brd4 binding to the PD-L1 promoter region to inhibit transcription (Zhu et al., 2016). By inhibiting eIF4E phosphorylation, eFT508 inhibits PD-L1 protein translation (Xu et al., 2019). Curcumin inhibits deubiquitinase CSN5 to promote PD-L1 ubiquitination degradation (Lim et al., 2016). However, most of these compounds are experimental tool drugs, which can’t realize the transformation of clinical application, and only stay in laboratory research. Besides, although previous studies have shown that other tyrosine kinases (such as Gefitinib (Lin et al., 2015) and Imatinib (Seifert et al., 2017; Hacking et al., 2022)) have been found to inhibit the transcription of PD-L1, our story uncovers another novel mechanism by tyrosinase inhibitors to reduce PD-L1 by targeting c-Myc.

c-Myc is one of the classical transcription factors of PD-L1, which is overexpressed in a variety of human malignant tumors (Nair and Burley, 2003; Han et al., 2012). c-Myc forms a heterodimer with Max and binds to the E-box sequence near the core promoter element of the active transcription gene, thereby over activating the transcription of the target gene (Blackwood and Eisenman, 1991). c-Myc acted as a general transcriptional amplifier (Lin et al., 2012), which bound to the promoter regions of the genes coding for PD-L1 (Casey et al., 2016). The stability of c-Myc depends on p-c-Myc (S62) and p-c-Myc (T58), which regulate the transcriptional activity of c-Myc (Farrell et al., 2013; Farrell and Sears, 2014). As one of the targets of Bafetinib (Kimura et al., 2005; Niwa et al., 2007), it has been reported that Abl could enhance T58/S62 phosphorylation of c-Myc, which is likely to lead to the increase of c-Myc transcriptional activity (Sanchez-Arevalo Lobo et al., 2013). We speculate that Bafetinib is likely to inhibit the transcription of c-Myc by targeting the activity of Abl thereby inhibiting the transcription of PD-L1. Although c-Myc usually leads to the occurrence and development of malignant tumors (Iwakawa et al., 2011), it is difficult to be targeted by traditional small molecule drugs because c-Myc is a natural disordered protein and lacks available drug recognition sites (Sorolla et al., 2020). Finding small molecule inhibitors that can target on c-Myc protein directly has been a major problem in drug research and development in the world for a long time. In this paper, our results show that Bafetinib can inhibit the expression of PD-L1 by reducing the transcription of c-Myc, both *in vivo* and *in vitro*. However, how Bafetinib inhibits the transcription of c-Myc and whether it affects the interaction between c-Myc and Max still needs further research.

Overall, our study shows that Bafetinib can inhibit the transcription of PD-L1 by reducing the transcription of c-Myc. Our findings provide an idea of PD-L1 in cancer cells and make it possible for small molecule inhibitors to target c-Myc protein directly. This strengthens the prospect of c-Myc as a drug target and provides a new idea for clinical immunotherapy.

## Data Availability

The original contributions presented in the study are included in the article/Supplementary Material, further inquiries can be directed to the corresponding authors.
