# Supplementary material for: Bafetinib Suppresses the Transcription of PD-L1 Through c-Myc in Lung Cancer
Source: Front Pharmacol. 2022 Jun 2;13:897747. doi: 10.3389/fphar.2022.897747 (PMC9201485; doi:10.3389/fphar.2022.897747)
Supplement: Supplementary file 1 [file DataSheet1.docx]

**Supplementary Figure 1**

**
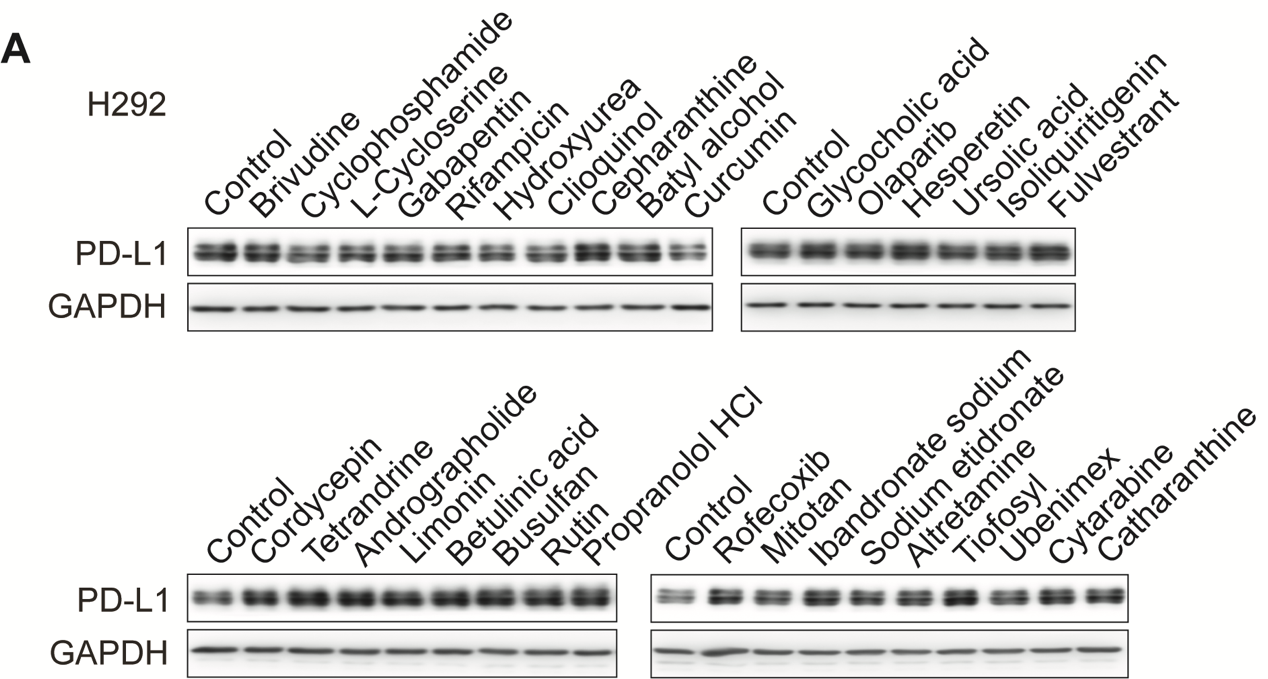
**

(A) Screening on H292 cells treated with different small molecule drugs (10μM) for 24 h.

**Supplementary Tables:**

| **Gene name** | | **Primer Sequence (5’—3’)** |
| --- | --- | --- |
| Negative control siRNA | | UUCUCCGAACGUGUCACGUTT |
| *MYC* | siRNA#1 | CCAUAAUGUAAACUGCCUCAA |

Table S1. siRNA sequences

| **Gene name** | **Sequence** | |
| --- | --- | --- |
| *CD274* | Forward | 5’-TCACTACACAGCCCTCCTAA-3’ |
|  | Reverse | 5’-ACACCAGAATATGGCCAAGAG-3’ |
| *MYC* | Forward | 5’-AACGATTCCTTCTAACAG-3’ |
|  | Reverse | 5’-GGCTAAATCTTTCAGTCT-3’ |
| *ACTIN* | Forward | 5’-ATTCCTATGTGGGCGACGAG-3’ |
|  | Reverse | 5’-CCAGATTTTCTCCATGTCGTCC-3’ |

Table S2. Human primers used in quantitative real time-PCR
